# Supplementary material for: Repression of TERRA Expression by Subtelomeric DNA Methylation Is Dependent on NRF1 Binding
Source: Int J Mol Sci. 2019 Jun 7;20(11):2791. doi: 10.3390/ijms20112791 (PMC6600276; doi:10.3390/ijms20112791)
Supplement: Supplementary file 1 [file ijms-20-02791-s001.pdf]

Figure S1

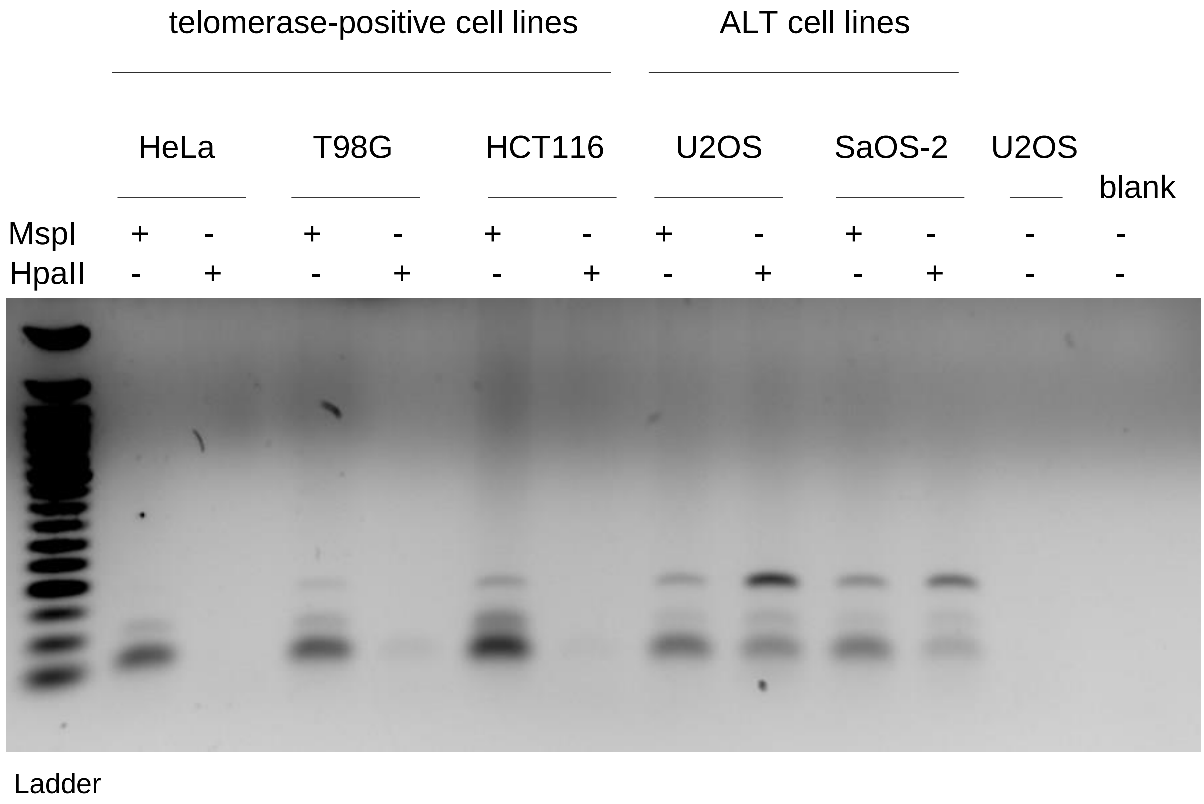

Figure S1. The level of methylation of the subtelomeric 29-bp repeats is lower in ALT cell lines than in telomerase-positive cell lines. Methylation of the 29-bp repeats in telomerase-positive and ALT cell lines was analyzed using the method described in Figure 1B: 500 ng of genomic DNA was digested with HpaII (methyl sensitive) or MspI (methyl insensitive), 1/20 of the elongation reaction was amplified by PCR (31 PCR cycles), and PCR products were run on a 1.5% BET-agarose gel. Undigested genomic DNA from U2OS cells and a sample without DNA (blank) were used as negative controls. Ladder is the 50-bp DNA ladder (New England BioLabs).

Figure S2

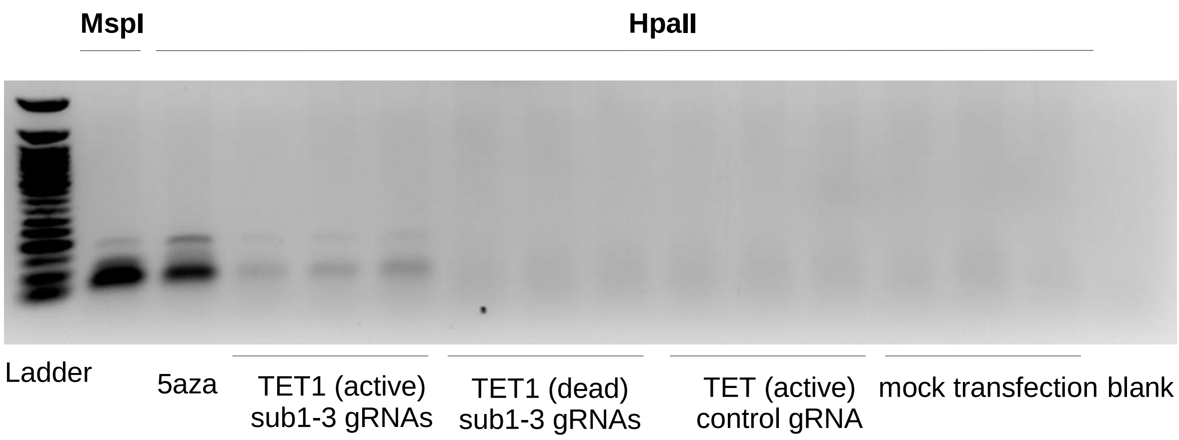

Figure S2. The demethylation of the 29-bp repeats induced by the CRISPR-dCas9-TET1 system is significantly weaker than the demethylation induced by 5-aza-dC treatment. Methylation of the 29-bp repeats in HeLa cells 96 hours after mock transfection or transfection with the CRISPR-dCas9-TET1 system and in HeLa cells treated with 5-aza-dC for 72 hours was analyzed using the method described in Figure 1B: 1 µg of genomic DNA was digested with HpaII, 1/20 of the elongation reaction was amplified by PCR (30 PCR cycles), and PCR products were run on a 1.5% BET-agarose gel. Each lane corresponds to an independent transfection experiment. Genomic DNA from untreated HeLa cells digested with MspI was used as positive control, and a sample without DNA (blank) was used as negative control. Ladder is the 50-bp DNA ladder (New England BioLabs).

Figure S3

NRF1 consensus	Y	G	C	G	C	A	Y	G	C	G	C	R
	80	80	85	90	100	95	75	95	100	100	100	85
TFB2M	T	T	C	G	C	A	T	G	C	G	C	A
29-bp repeat (1)	T	G	C	G	C	C	T	G	C	G	C	C
29-bp repeat (2)	T	G	C	G	C	C	G	G	C	G	C	G

Figure S3. The NRF1 binding sites located in the 29-bp repeats differ by two nucleotides from the NRF1 consensus binding site. Sequences of the NRF1 consensus binding site, of the sites located at the *TFB2M* promoter, and of the two sites located in the 29-bp repeats are indicated. Numbers below the consensus represent the percent presence of the indicated nucleotide at that position in 20 functional NRF1 binding sites. Y indicates pyrimidine nucleotide, R indicates purine nucleotide. Nucleotides that differ from the consensus are in red.

Figure S4

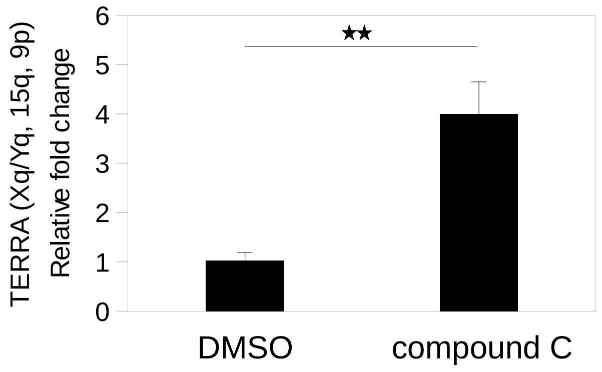

Figure S4. Compound C induces an increase of TERRA levels in HCT116 cells. RT-qPCR analysis of TERRA levels (Xq, Yq, 15q, 9p) in HCT116 cells treated with DMSO or compound C (5 µM) for 18 hours. TERRA was quantified by RT-qPCR using TR and TF primers; levels were normalized to *GAPDH* mRNA, and all values were compared to DMSO-treated sample. The bars represent the average values from three biological and two technical replicates for each sample. Error bars represent the standard deviations. P values were calculated by paired two-tailed Student's t-test (n=3). $^{**}P<0.01$.
